# The theory of planned behaviour and discrete food choices: a systematic review and meta-analysis

**DOI:** 10.1186/s12966-015-0324-z

**Published:** 2015-12-30

**Authors:** Máirtín S. McDermott, Madalyn Oliver, Alexander Svenson, Thomas Simnadis, Eleanor J. Beck, Tim Coltman, Don Iverson, Peter Caputi, Rajeev Sharma

**Affiliations:** School of Computing and Information Technology, Faculty of Engineering and Information Sciences, University of Wollongong, Wollongong, NSW 2522 Australia; School of Medicine, Faculty of Science, Medicine and Health, University of Wollongong, Wollongong, NSW 2522 Australia; School of Management, Operations and Marketing, Faculty of Business, University of Wollongong, Wollongong, NSW 2522 Australia; Faculty of Health, Arts and Design, Swinburne University of Technology, Melbourne, VIC 3122 Australia; School of Psychology, Faculty of Social Sciences, University of Wollongong, Wollongong, NSW 2522 Australia; Centre for Health and Social Research (CHaSR), Australian Catholic University, Melbourne, Victoria Australia

**Keywords:** Discrete food choice, Theory of reasoned action, Theory of planned behaviour, Systematic review, Meta-analysis

## Abstract

The combination of economic and social costs associated with non-communicable diseases provide a compelling argument for developing strategies that can influence modifiable risk factors, such as discrete food choices. Models of behaviour, such as the Theory of Planned Behaviour (TPB) provide conceptual order that allows program designers and policy makers to identify the substantive elements that drive behaviour and design effective interventions. The primary aim of the current review was to examine the association between TPB variables and discrete food choice behaviours. A systematic literature search was conducted to identify relevant studies. Calculation of the pooled mean effect size (*r*_*+*_) was conducted using inverse-variance weighted, random effects meta-analysis. Heterogeneity across studies was assessed using the Q- and I^2^-statistics. Meta-regression was used to test the impact of moderator variables: type of food choice behaviour; participants’ age and gender. A total of 42 journal articles and four unpublished dissertations met the inclusion criteria. TPB variables were found to have medium to large associations with both intention and behaviour. Attitudes had the strongest association with intention (*r*_*+*_ = 0.54) followed by perceived behavioural control (PBC, *r*_*+*_ = 0.42) and subjective norm (SN, *r*_*+*_ = 0.37). The association between intention and behaviour was *r*_*+*_ = 0.45 and between PBC and behaviour was *r*_*+*_ = 0.27. Moderator analyses revealed the complex nature of dietary behaviour and the factors that underpin individual food choices. Significantly higher PBC-behaviour associations were found for choosing health compromising compared to health promoting foods. Significantly higher intention-behaviour and PBC-behaviour associations were found for choosing health promoting foods compared to avoiding health compromising foods. Participant characteristics were also found to moderate associations within the model. Higher intention-behaviour associations were found for older, compared to younger age groups. The variability in the association of the TPB with different food choice behaviours uncovered by the moderator analyses strongly suggest that researchers should carefully consider the nature of the behaviour being exhibited prior to selecting a theory.

**Electronic supplementary material:**

The online version of this article (doi:10.1186/s12966-015-0324-z) contains supplementary material, which is available to authorized users.

## Background

Non-communicable diseases such as cardiovascular disease, cancers and diabetes accounted for over two thirds of global deaths in 2012 [1] and are predicted to account for up to US$46.7 trillion in cumulative losses in productivity between 2011 and 2030 [2]. These combined economic and social costs provide a compelling argument for developing strategies to address the key modifiable risk factors of these conditions, such as dietary consumption [3]. Domestic health authorities [4–6] are aware of the importance of encouraging healthy eating patterns and have developed guidelines that seek to optimise the health and wellbeing of their citizens. These guidelines have a focus on eating ‘core’ food groups, such as vegetables, fruits and whole-grains while avoiding the consumption of foods that are classified as ‘non-core’ or ‘discretionary’, such as cakes, pastries and soft drinks.

The healthy eating guidelines are reflective of a broader shift within the discipline of nutrition, away from the effects of nutrients to a more holistic notion of the effects of specific foods in the context of a whole diet [7]. This transition aligns with the premise that discrete ‘food’ choices, i.e. those decisions made by individuals at the point of consumption, rather than ‘nutrient’ choices drive eating patterns. These decisions are of particular interest due to the persistence of poor food choices within the general population (e.g. [8]). The challenge is to encourage health promoting behaviours, such as choosing whole over refined grain cereal products, increasing fruit and vegetables and consciously limiting discretionary food intake in order to reduce the risk of chronic disease.

Understanding the underpinnings of these behaviours will provide invaluable assistance in the development of behaviour change interventions, which are more likely to be effective when based on sound theory [9]. The Theory of Planned Behaviour (TPB) [10], which is an extension of the earlier Theory of Reasoned Action (TRA) [11] is one of the models most commonly used to understand health behaviours such as these. The TPB asserts that the most proximal determinant of behaviour is the *intention* to perform that behaviour. Intentions, which are considered to indicate the amount of effort an individual is likely to devote to performing a behavior, are in turn determined by attitudes, an overall evaluation of the behaviour, subjective norms (SN), an evaluation of whether an individual feels significant others think he/she should engage in the behaviour and perceived behavioural control (PBC), which represents an individual’s perceptions of control over that behaviour. To the extent that it reflects actual control, PBC is also held to exert a direct effect on behaviour. Thus, according to the theory, individuals will have a strong intention to, for example, eat the recommended daily amount of vegetables, when they hold positive attitudes towards that behaviour, perceive social pressures from those whose opinions they value and feel capable of eating the recommended amount without difficulty. This intention, along with their perceptions of capability, determines the likelihood that they will perform this behaviour. The effect of all other influences, for example biological, environmental and cultural, are hypothesised to be mediated by the TPB [12].

The potential of the TPB as a model for understanding health behaviours was confirmed by a review conducted by McEachan et al. [13] which found that the theory accounted for between 14 and 24 % of the between-study variance in behaviour. However, this review combined studies examining discrete food choice behaviours such as eating fruit and vegetables or avoiding sugar-sweetened drinks, with broader dietary patterns such as ‘eating a healthy diet’. Thus, their analyses may have masked important differences. For example, previous research has found that the association between TPB variables and behaviour may differ between broad categories of behaviour such as ‘healthy eating’ and more specific behaviours, such as food choices [14]. These data are also lacking when considered from a clinical perspective. Although dietary guidelines provide an overview of broad dietary patterns that will achieve optimum health for populations, clinicians are faced with the difficult task of managing the myriad of discrete food choices that shape these eating patterns. A more nuanced examination of the literature is therefore warranted. The primary aim of the current study is to examine the association between TPB variables and discrete food choice behaviours.

This review also has a number of secondary aims. Primarily, these aim to elucidate the association between the TPB and food choice behaviours by examining whether associations are moderated by the *type* of food choice behaviour examined. Critics argue that TPB research is frequently applied to a broad range of behaviour without consideration of whether such application is valid given the *nature* of the behaviour in question. The TPB is generally considered to present a rational view of behaviour, determined largely through a process of deliberative appraisal. When examined from alternative theoretical perspectives, the extent to which these processes apply to some food choices is open to question.

For example, from the perspective of Temporal Self-Regulation Theory (TST) [15], the TPB is deficient for not considering the differential temporal weighting of anticipated contingencies of health behaviours. Health-compromising behaviours, such as choosing highly palatable, calorie dense or high-fat foods, are frequently associated with many benefits and few costs at the point of action, whereas the same behaviours are associated with long term costs and few if any long term benefits. In contrast, health-promoting behaviours, such as choosing lower energy density, nutrient rich foods, frequently work in the opposite manner. In line with TST, the strength of association between the deliberative processes captured by the TPB and behaviour is hypothesised to vary based on the temporal frame of the specific food choice [16, 15].

Our second set of analyses will examine the literature from the perspective of dual-process models of behaviour (e.g. [17, 18]). These models propose that behaviour is determined by two interacting, parallel systems whereby automatic, impulsive processes, are in competition with more deliberative, rational determinants of behaviour such as those described in the TPB. Given that health promoting food choices can be broadly categorised into one of two groups: (a) choosing health promoting foods that typically have a lower hedonic value than alternative foodstuffs, and may therefore be governed by deliberative processes; and (b) resisting the impulse to choose health compromising foods with high hedonic value, one might expect the association between the TPB and behaviour to be stronger for (a) than (b). In order to inform the development of interventions facilitating health-promoting food choices, we shall also examine this distinction here.

Finally, in order to generate further evidence that may facilitate the development of targeted interventions, we will also examine the impact of participant characteristics within the context of health promoting food choice behaviours. Specifically we will examine whether the associations specified within the TPB are moderated by age and gender. National survey data from Australia [8] has found important differences in food choices based on these characteristics. For example, that men are more likely to consume unhealthy foods such as soft drinks or burgers, and that that teenagers and younger adults are less likely to consume fruit compared to the general population, a pattern that is repeated worldwide [19].

## Review

### Methods

The design, conduct and reporting of this systematic review was informed by the Preferred Reporting Items for Systematic Reviews and Meta-Analyses guidelines (PRISMA [20]) (the PRISMA checklist is available as Additional file 1). As the study involved the secondary analysis of existing datasets, ethical approval was not sought. The funding organization for this study had no role in the collection, analysis and interpretation of data, or the right to approve the finished manuscript prior to publication. As the current study was conducted as part of a larger program of research, no study protocol was produced.

### Selection criteria

In accordance with PRISMA, the PICOS (population, intervention, comparison, outcome, study design) approach [21] was used to formulate the selection criteria. Studies that explicitly applied the TRA or TPB to the choice of specific foods (e.g. high fibre bread, fruit, fish) or narrow categories of foods (e.g. high calorie snacks, dairy products, ready meals) were included. Studies investigating associations between these models and dietary patterns (e.g. healthy eating or eating a low-fat diet) were not included in the current review. These have been examined elsewhere [22]. Studies were included where participants were drawn from a **population** without any current or former psychiatric or medical condition, for example eating disorders or diabetes, as the psychological determinants of food choice behaviours in these populations may not be generalizable to the community at large. In line with previous reviews (e.g. [23]), studies were excluded if participants received an **intervention.** Studies were not selected based on any **comparison** between conditions. In line with theoretical models, TRA studies must at minimum have reported correlations between the following **outcomes**: attitudes and subjective norm with intentions, and intentions with behaviour. TPB studies must at minimum have reported correlations between attitudes, subjective norm and perceived behavioural control with intentions, and perceived behavioural control and intentions with behaviour. We included any quantitative **study design** provided the other inclusion criteria were met. In addition, studies needed to report sample size, full details (i.e. item wording, response scale and response anchor) of at least one of the items used to measure each variable and be published in the English language.

### Study identification

We searched for published and unpublished research in PsycINFO, MEDLINE, Web of Science, CINAHL and ProQuest Dissertations & Theses. Full details of the electronic search strategies used can be found in Additional file 2. We also manually searched the reference lists of all studies selected for inclusion, and the reference list of a key systematic review [13]. Final searches were conducted in October 2014.

Two authors (MO & MSMcD) pre-screened one half of the database containing all titles and abstracts for relevance. These studies were then selected for inclusion independently by the same two authors. Agreement was substantial (κ = .80) [24]. Data from each study was extracted by one of two authors (MO, AS) who also independently coded each effect size for behaviour type. Again, agreement was substantial (κ = .72).

### Data extraction

In addition to correlations and sample size, we extracted data on gender, the mean age of participants, and the behaviour examined in each study. In order to test for the impact of temporal frame, each behaviour was coded as *health promoting* or *health compromising*. The health promoting category included studies where the food choice was deemed to have few benefits and potential costs (e.g. bland or unpleasant taste) at the point of action, but with benefits and few costs in the long term (e.g. reduction in risk for contracting chronic conditions such as coronary heart disease). Examples of behaviours coded into this category include choosing fruits and vegetables, high fibre bread and dairy products. The health compromising category contained studies examining behaviours that operated in the opposite manner, for example, choosing soft drinks, sugared snacks or junk food. Studies in which the temporal frame of the target behaviour was not clear were not included in these analyses.

Subsequently, we coded each health promoting behaviour into two further categories depending on whether the behaviour targeted *choosing* health promoting, or *avoiding* health compromising foods. Each decision was made based primarily on an examination of the items used to evaluate TRA/ TPB variables. For example, the study conducted by De Bruijn & Van den Putte [25] assessed the consumption of soft drinks as the behavioural outcome (e.g. how many days per week do you consume sugar-sweetened soft drinks?), but the behaviour targeted by the cognitive items was to limit consumption of soft drinks (e.g. I intend to limit my amount of soft drink consumption’). The ‘target behaviour’ was therefore coded as avoiding the consumption of health compromising foods. A descriptive summary of each of the studies included in the review, including target behaviour and the coding each behaviour received can be found in Table 1. A glossary of key terms and definitions used in the review is provided in Additional file 3.

**Table 1:** Descriptive summary of studies included in the systematic review (*n* = 43)

Study	Article Type	Country	N	Gender	Age Category	Theory	Food choice	Behaviour coding
Aghamolaei et al. (2012) [43]	Journal article	Iran	321	62.9 %	≥30	TPB	Fish	NA
Astrom (2004) [44]/Astrom & Okullo (2004) [29]	Journal article	Uganda	1146	Not specified	≤17	TRA	Sugared snacks	Choosing health compromising food
Balian (2008) [45]	Dissertation	USA	93	54.0 %	≤17	TPB	Milk, soda	Choosing health compromising food (Soda only)
Berg et al. (2000) [30]/Conner et al. (2011) [31]	Journal article	Sweden	1096	52.0 %	≤17	TPB	Milk, high fibre bread	Choosing health promoting food (High fibre bread only)
Blanchard et al. (2009a) [46]	Journal article	USA	511	49.7 %	18-29	TPB	Fruit & vegetables	Choosing health promoting food
Blanchard et al. (2009b) [47]	Journal article	USA	176-237	56.9 %	18-29	TPB	Fruit & vegetables	Choosing health promoting food
Branscum & Sharma (2014) [48]	Journal article	USA	69-98	100.0 %/ 0.0 %	≤17	TPB	Snack foods, fruit & vegetables	Avoiding health compromising food (snacks), choosing health promoting food (fruit & vegetables)
Brug et al. (2006) [49]	Journal article	The Netherlands	627	50.9 %	≥30	TPB	Fruit	Choosing health promoting food
Churchill et al. (2008) [50]	Journal article	UK	315	65.7 %	≥30	TPB	High-calorie snacks	Avoiding health compromising food
Churchill & Jessop (2011) [51]	Journal article	UK	139	77.7 %	18-29	TPB	High-calorie snacks	Choosing health compromising food
Collins & Mullan (2011) [16]	Journal article	Australia	190	77.9 %	18-29	TPB	Snacks, fruit & vegetables	Choosing health compromising food (snacks), choosing health promoting food (fruit & vegetables)
Corry (2008) [52]	Dissertation	USA	159	53.5 %	18-29	TPB	Fruit & vegetables	Choosing health promoting food
De Bruijn (2010) [53]	Journal article	The Netherlands	538	53.7 %	≥30	TPB	Fruit	Choosing health promoting food
De Bruijn et al. (2012) [54]	Journal article	The Netherlands	159	78.0 %	18-29	TPB	Fruit	Choosing health promoting food
De Bruijn & Van den Putte (2009) [25]	Journal article	The Netherlands	312	65.3 %	≤17	TPB	Soft drinks	Avoiding health compromising food
De Bruijn et al. (2009) [55]	Journal article	The Netherlands	405	57.1 %	≥30	TPB	Fruit	Choosing health promoting food
De Bruijn et al. (2007a) [56]	Journal article	The Netherlands	521	53.7 %	≥30	TPB	Fruit	Choosing health promoting food
De Bruijn et al. (2007b) [57]	Journal article	The Netherlands	208	62.0 %	≤17	TPB	Soft drinks	Avoiding health compromising food
De Bruijn et al. (2005) [58]	Journal article	The Netherlands	3859	55.2 %	≤17	TPB	Snacks	Avoiding health compromising food
Karimi-Shahanjarini et al. (2012) [59]	Journal article	Iran	790	100.0 %	≤17	TPB	Junk food	Choosing health promoting food
Kassem (2000) [32]/Kassem et al. (2003) [33]	Dissertation/Journal article	USA	710	100.0 %	≤17	TPB	Milk, soft drinks	Choosing health compromising food (for soft drinks only)
Kassem & Lee (2004) [60]	Journal article	USA	564	0.0 %	≤17	TPB	Soft drinks	Choosing health compromising food
Kassem & Lee (2005) [61]	Journal article	USA	560	0.0 %	≤17	TPB	Milk	NA
Kida & Astrom (1998) [62]	Journal article	Tanzania	309	46.5 %	≤17	TPB	Sugared snacks	Avoiding health compromising food
Kim et al. (2003) [63]	Journal article	USA	162	76.0 %	≥30	TPB	Dairy products	Choosing health promoting food
Mahon et al. (2006) [64]	Journal article	UK	1004	86.0 %	Not specified	TPB	Ready meals, takeaways	Choosing health compromising food (ready meals & takeaways)
Masalu & Astrom (2001) [65]	Journal article	Tanzania	1090	32.0 %	18-29	TPB	Sugared snacks	Avoiding health compromising
Mitterer-Daltoe et al. (2013) [66]	Journal article	Brazil	200	60.0 %	≥30	TPB	Fish	NA
Murnaghan et al. (2010) [67]	Journal article	Canada	287	51.0 %	≤17	TPB	Fruit & vegetables	Choosing health promoting food
O’Neal et al. (2014) [68]	Journal article	USA	211	73.0 %	≥30	TPB	Fruit & vegetables	Choosing health promoting food
Onwezen et al. (2014) [69]	Journal article	The Netherlands	491	50.0 %	≥30	TPB	Fruit	Choosing health promoting food
Povey et al. (2000) [14]	Journal article	UK	151	70.0 %	≥30	TPB	Fruit & vegetables	Choosing health promoting food
Prell et al. (2002) [70]	Journal article	Sweden	162	53.3 %	≤17	TPB	Fish	NA
Richetin et al. (2008) [71]	Journal article	UK	75	69.4 %	18-29	TPB	Soft drinks	Choosing health compromising food
Sharifirad et al. (2013) [72]	Journal article	Iran	521	46.8 %	≤17	TPB	Fast food	Choosing health compromising food
Sjoberg et al. (2012) [73]	Journal article	USA	258	80.6 %	≥30	TPB	Wholegrain bread	Choosing health promoting food
Tak et al. (2011) [74]	Journal article	The Netherlands	970	46.0 %	≤17	TPB	Sugar-sweetened beverages	Choosing health compromising food
Tak et al. (2013) [75]	Journal article	The Netherlands	323	54.1 %	≥30	TPB	Fruit	Choosing health promoting food
Towler & Shepherd (1991) [76]	Journal article	UK	288	61.5 %	≥30	TRA	Chips	Choosing health compromising food
Tuu et al. (2008) [77]	Journal article	Vietnam	612	59.3 %	≥30	TPB	Fish	NA
Verbeke & Vackier (2005) [78]	Journal article	Belgium	429	66.9 %	≥30	TPB	Fish	NA
Verplanken (2006) [79]	Journal article	Norway	128	64.1 %	Not specified	TPB	Snacks	Choosing health compromising food
Zoellner et al. (2012) [80]	Journal article	USA	119	66.0 %	≥30	TPB	Sugar-sweetened beverages	Avoiding health compromising food

*Notes:* Gender was coded as the proportion of the sample that was female; Behaviour coding is provided for the behaviour targeted in each study. These were coded either as one of two types of health promoting food choice behaviour (choosing vs avoiding), or as a health compromising food choice behaviour. Behaviours coded as not applicable (NA) were not included in these analyses

A number of studies included in each meta-analysis provided multiple effect sizes that were eligible for inclusion. The decision of how to handle these data was guided by Borenstein et al. [26] and Sharma et al. [27]. In instances where multiple effect sizes were due to data being presented for independent samples or where data was presented from two or more time points using the same participants, these were treated as individual data points for analysis. Where multiple measurements of behaviour eligible for inclusion in the meta-analysis were reported in the same study, each was retained provided it yielded distinct information. Where data for ‘higher order’ TPB constructs (e.g. instrumental and descriptive norms) or individual items for constructs were reported separately, these were clustered to yield single correlations.

### Data analysis

Calculation of the pooled mean effect size (*r*_*+*_) was conducted using inverse-variance weighted random effects meta-analysis [26]. We also estimated the heterogeneity across studies, using both the *Q* and *I*^*2*^ statistics. To test for moderation we employed the protocol for random effects meta-regression recommended by Borenstein et al. [26]. All analyses were performed using Comprehensive Meta-Analysis (CMA) Version 3.0 [28].

## Results

### Search results

**Fig. 1:**
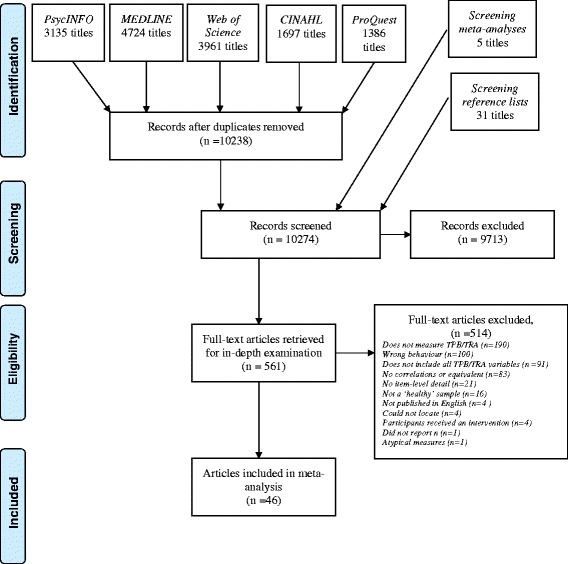
PRISMA flow chart

The electronic search strategy retrieved 10,238 unique records. A further five were identified through screening the reference lists of a related meta-analysis [13] and 31 through screening the reference lists of included articles. In total, 42 journal articles and four dissertations met the inclusion criteria. Data from three studies were reported in more than one article ([29], [30, 31] and [32, 33]). Relevant data were extracted from either article as appropriate. A total of 43 studies were therefore included. Full details of the screening process can be seen in the PRISMA Flow-Chart (Fig. 1).

### Association of TPB variables with intention and food choice

Table 1 summarises the meta-analysed associations for TPB variables across studies. TPB variables were found to have medium to large associations with both intention and behaviour. Attitudes had the strongest association with intention (*r*_*+*_ = 0.54) followed by PBC (*r*_*+*_ = 0.42) and SN (*r*_*+*_ = 0.37). The association between intention and behaviour was *r*_*+*_ = 0.45 and between PBC and behaviour was *r*_*+*_ = 0.27. Forest plots for each association can be found in Additional file 4. Both the Q- and *I*^*2*^-statistics revealed significant heterogeneity for each effect size (see Table 2) supporting the use of meta-regression to search for moderators.

**Table 2:** Random-effects average correlation and heterogeneity statistics for TPB associations and healthy eating

Association	*n*	*k*	*r*_*+*_	CI	*Q*	*I* ^*2*^
Attitude-intention	28572	54	0.54	0.49-0.58	1202.19^***^	95.59
Subjective norm-intention	28572	54	0.37	0.33-0.42	988.66^***^	94.63
PBC-Intention	28284	53	0.42	0.36-0.48	2015.34^***^	97.42
PBC-Behaviour	28737	54	0.27	0.23-0.32	843.16^***^	93.71
Intention-Behaviour	29465	60	0.45	0.40-0.49	1326.56^***^	95.55

*n* = number of participants, *k* = number of effect sizes included in the analysis, CI = 95 % confidence interval, *Q* and *I* ^*2*^ = tests of heterogeneity, *r*_*+*_ = random effects average correlation, ^***^ *p* < .001

### Test for moderation by behaviour type

Mean effect sizes and heterogeneity statistics for each category of behaviour are shown in Table 3. As we were interested in the association between these behaviour types and assessments of behaviour, analyses were limited to intention-behaviour, and PBC-behaviour associations. The first analysis compared health promoting with health compromising food choices from the perspective of TST. There was no evidence of moderation for the intention-behaviour association (*r*_*+*_ = 0.38 and 0.45, *χ*^*2*^(1) = 2.59, *n.s.*). However, there was a significant difference between health promoting and health compromising food choices for the PBC-behaviour association (*r*_*+*_ = 0.31 and 0.17, *χ*^*2*^(1) = 8.23, *p* < 0.01).

**Table 3:** Random-effects average correlation and heterogeneity statistics by category of behaviour

	Health promoting food choice behaviours								Health compromising food choice behaviours			
	Consume health promoting				Avoid health compromising							
Association	*n*	*k*	*r*_*+*_	*I* ^*2*^	*n*	*k*	*r*_*+*_	*I* ^*2*^	*n*	*k*	*r*_*+*_	*I* ^*2*^
PBC-Behaviour	6413	19	0.35	92.56	6232	7	0.21	87.71	8726	17	0.17	93.22
Intention-Behaviour	7676	21	0.43	87.98	6518	10	0.28	90.16	9013	18	0.45	94.87

*n* = number of participants, *k* = number of effect sizes included in the analysis, CI = 95 % confidence interval, *I* ^*2*^ = tests of heterogeneity, *r*_*+*_ = random effects average correlation

Following this, we tested whether there was evidence of moderation *within* health promoting food choices. On this occasion there was evidence of moderation for both the PBC-behaviour (*r*_*+*_ = 0.35 and 0.21, *χ*^*2*^(1) = 4.38, *p* < 0.05) and intention-behaviour associations (*r*_*+*_ = 0.43 and 0.28, *χ*^*2*^(1) = 7.93, *p* < 0.01) for choosing health promoting and avoiding of health compromising food, respectively.

### Test for moderation by participant characteristics

Table 4 shows the breakdown of effect sizes by mean age of participants. Age moderated the intention-behaviour association (*χ*^*2*^(2) = 9.22, *p =* 0.01), with the ≤17 age group differing significantly from the 30+ age group (*B* = −0.21 [95 % CI: −0.35, −0.06] *p* < 0.01). There was no evidence that age moderated any of the other associations in the model. Gender was found not to moderate any of the associations within the TPB.

**Table 4:** Random-effects average correlation and heterogeneity statistics by age of participants

	≤17				18-29				≥30			
Association	*n*	*k*	*r*_*+*_	*I* ^*2*^	*n*	*k*	*r*_*+*_	*I* ^*2*^	*n*	*k*	*r*_*+*_	*I* ^*2*^
Attitude-intention	6238	8	0.46	93.13	3149	9	0.41	71.52	3583	11	0.55	92.82
SN-intention	6238	8	0.35	95.69	3149	9	0.32	85.62	3583	11	0.33	82.86
PBC-Intention	6238	8	0.41	95.63	3149	9	0.55	95.50	3583	11	0.45	91.62
PBC-Behaviour	5975	6	0.21	92.39	3213	10	0.34	89.07	3475	10	0.35	91.70
Intention-Behaviour	6309	10	0.26	92.68	3213	10	0.42	74.86	3576	11	0.44	89.08

*n* = number of participants, *k* = number of effect sizes included in the analysis, CI = 95 % confidence interval, *I* ^*2*^ = tests of heterogeneity, *r*_*+*_ = random effects average correlation. NB Restricted to *health promoting* food choice behaviours only

## Discussion

To our knowledge, the current review is the first to examine the associations between TPB variables and food choice behaviours. In general, these associations were medium (SN and PBC with intention, PBC and intention with behaviour) and large (attitude with intention) in magnitude. When considered together, the associations between TPB variables and food choice behaviours were similar to those reported by McEachan et al. [13]. However, when individual behaviours were coded into distinct categories, we found differences with important theoretical and practical implications.

### Moderation by type of food choice behaviour

As per TST, one might have expected the intention to choose highly palatable, energy-dense foods to have stronger associations with behaviour, as these food choices are typically supported by strong positive immediate contingencies to consume compared to ‘healthy’ food choices where immediate contingencies are few [15]. However, when we examined differences between these behaviours from the perspective of TST, we failed to find an expected difference for the intention-behaviour association. We did, however, find that health promoting foods had a significantly higher PBC-behaviour association than health compromising foods. According to the TPB, PBC influences behaviour directly to the extent that it reflects actual control [34]. It may be, therefore, that people have inaccurate perceptions of their control over their decision to consume health compromising foods. Alternatively, given how easy it is for people to make health compromising food choices due to supportive immediate contingencies, it could be that perceptions of control are less relevant. This finding stands in contrast with previous research that has reported negative PBC-behaviour associations for health compromising behaviours [23, 35].

When health promoting foods were examined from the perspective of dual-process models of behaviour, we found that there was evidence of moderation both for the PBC-behaviour and intention-behaviour associations. In both cases, mean correlations were significantly larger for those studies examining choosing health promoting foods, as opposed to avoiding health compromising foods. This suggests that the determinants of avoiding health compromising foods are less well captured by reflective, self-report measures than behaviours related to more deliberate, goal-oriented behaviour such as choosing foods in order to achieve health gains. Resisting the impulse to eat highly palatable, health compromising foods is likely to be explained better using assessment strategies designed to tap into non-reflective determinants of behaviour, for example using the Implicit Association Test (IAT, [36]).

### Moderation by participant characteristics

Intentions were more weakly associated with behaviour for those participants who were aged 17 or younger compared to the oldest age group. This mirrors the results reported for dietary behaviours by McEachan et al. [13], who also found PBC to have stronger associations with behaviour in older compared to younger age groups, a trend not apparent here. Previous studies have suggested that the TPB may be more weakly associated with food choice and other consumption behaviours in younger adults due to their being more likely to live at home and thus less likely to fully determine what they eat or drink (e.g. [23, 37]). We did not find any evidence that associations were moderated by participants’ gender. This is in spite of research suggesting that men and women differ in their dietary patterns [8, 19]. It is possible that our method of testing for the effects of gender, using the proportion of female participants in the sample, may have masked any true differences. It may instead have been preferable to use the method of Cooke et al. [23] who looked at differences between all-male, all-female and combined samples, however there were insufficient studies to conduct the analysis in this way (see Table 1). Further examination of this issue may be warranted.

### Strengths and limitations

Strengths of the current review include the broad search strategy, targeting both published and unpublished research and the use of established criteria [21] to guide the design, conduct and reporting of the meta-analysis. The review is limited, however, by the high level of heterogeneity reported for all associations. This is in spite of the inclusion of a sample of studies that were largely homogenous in terms of participants, behaviour and theoretical background. Finally, there is a further limitation in that the assessment of behaviour within many of the studies examined is crude, with very few studies recording actual dietary intake (see Additional file 5).

## Practical implications

Although the medium-to-large mean associations between TPB variables and behaviour identified in the current review suggest this model may provide a solid foundation for interventions to increase rates of health promoting food choices, any recommendation must be accompanied by some important caveats.

First, the current analyses strongly suggest that the rational variables captured by the TPB are more strongly associated with some food choices than others. It is imperative that researchers carefully consider the nature of the behaviour under examination prior to selecting a theory to predict that behaviour. The concept of a behavioural diagnosis is central to key frameworks for understanding the determinants of behaviour to inform intervention design [38]. The same considerations should be applied when selecting theory to use in observational research seeking to understand health behaviours.

Second, designers of interventions aiming to reduce the consumption of unhealthy foods should consider alternative approaches not reliant on changing rational determinants of behaviour such as those described in the TPB. Potential approaches include negative evaluative conditioning, training to withhold responses to tempting stimuli and the formation of implementation intentions [39, 40]. These interventions aim to help individuals counter the automatic impulses to consume that can arise when highly palatable, health compromising foods are encountered. It is worth noting, however, that even in cases where strong associations between the TPB and behaviour are suggested by the current review (e.g. with health promoting food choices), the utility of the model as a basis for developing effective interventions has not been supported by the evidence [41].

Third, it is important to remember that whilst the evidence presented here are informative, ultimately they provide an incomplete record of the potential of the TPB as a medium for behaviour change. This is due primarily to two reasons: (1) that the associations revealed in observational studies are not always reflected in experimental research [42]; and (2) the observational nature of the findings presented here leave open the possibility that the relationship is spurious and that an unmeasured variable has a causal impact on both TPB variables and behaviour. Experimental research aiming to facilitate a change in food choice behaviours by targeting TPB variables is clearly warranted.

## Conclusions

There is a clear imperative for the design of interventions to maximise the health and wellbeing populations by increasing rates of health promoting food choices and concurrently discouraging health-compromising food choices. Understanding the key determinants of these behaviours can assist in the development of such interventions. Based on the evidence presented here, the potential of the TPB as a model either to understand these behaviours or serve as a basis for intervention development, appears limited. Although we found that TPB variables had medium to large associations both with intention and behaviour, the associations between key variables and behaviour were significantly lower both for choosing and avoiding health compromising foods, and in younger age groups. The current review reinforces the complex nature of dietary behaviour and the factors that underpin individual food choices and highlights the need to consider alternative models and determinants of behaviour.

## Additional files

Additional file 1:
**PRISMA Checklist.** (DOCX 26 kb) Additional file 2:
**Electronic search strategies.** (DOCX 13 kb) Additional file 3:
**Glossary of key terms and definitions.** (DOCX 21 kb) Additional file 4:
**Forest plots for associations between TRA/TPB variables and behaviour.** (DOCX 541 kb) Additional file 5:
**Details of method used to measure behaviour in each study.** (DOCX 19 kb)
