# Supplementary material for: The theory of planned behaviour and discrete food choices: a systematic review and meta-analysis
Source: Int J Behav Nutr Phys Act. 2015 Dec 30;12:162. doi: 10.1186/s12966-015-0324-z (PMC4696173; doi:10.1186/s12966-015-0324-z)
Supplement: Additional file 2: — Electronic search strategies. (DOCX 13 kb) [file 12966_2015_324_MOESM2_ESM.docx]

**Additional File 2: Electronic search strategies**

**PsycINFO & MEDLINE**

1. ("theory of planned behav*" OR "theory of reasoned action" OR intent*).mp.
2. (("perceived behavioural control" OR "perceived behavioral control" OR "subjective norm*" OR "attitude*") AND intent*).mp.
3. (eat* or diet* or consumption).mp.
4. (food or fruit* or vegetable* or fat or fibre or fiber or sugar* or snack*).mp.
5. 1 OR 2
6. 3 OR 4
7. 5 AND 6

**CINAHL**

1. ("theory of reasoned action" OR "theory of planned behav*" OR intent*)
2. (("perceived behavioural control" OR "perceived behavioral control" OR "subjective norm*" OR "attitude*") AND intent*)
3. (eat* OR diet* OR consumption OR food or fruit* or vegetable* or fat or fibre or fiber or sugar* or snack*)
4. S1 or S2
5. S4 AND S5

**WoS**

1. TS=("theory of reasoned action" OR "theory of planned behavior" OR "theory of planned behaviour")
2. TS=intention*
3. TS=(("perceived behavioural control" OR "perceived behavioral control" OR "subjective norm*" OR "attitude*") AND intention*)
4. TS=(eat* OR diet* OR consumption OR food or fruit* or vegetable* or fat or fibre or fiber or sugar* or snack*)
5. (#1 OR #2 OR #3) AND #4

**ProQuest Dissertations & Theses**

ab(eat* OR diet* OR consumption OR food OR fruit* OR vegetable* OR fat OR fibre OR fiber OR sugar* OR snack*) AND (ab(intention*) OR ab(("theory of reasoned action" OR "theory of planned behavior" OR "theory of planned behaviour")) OR ab((("perceived behavioural control" OR "perceived behavioral control" OR "subjective norm*" OR "attitude*") AND intention*)))
