# Supplementary material for: The theory of planned behaviour and discrete food choices: a systematic review and meta-analysis
Source: Int J Behav Nutr Phys Act. 2015 Dec 30;12:162. doi: 10.1186/s12966-015-0324-z (PMC4696173; doi:10.1186/s12966-015-0324-z)
Supplement: Additional file 3: — Glossary of key terms and definitions. (DOCX 21 kb) [file 12966_2015_324_MOESM3_ESM.docx]

**Additional File 3: Glossary of key terms and definitions**

| **Variable** | **Description** | **Sample measures** |
| --- | --- | --- |
| Attitude | Reflects the degree to which a person has a favourable or unfavourable evaluation of the behaviour in question ^1^. | Overall I think eating the recommended fruit & vegetables is… good/ bad.  I believe consuming a limited amount of soft drink is… very pleasant/ very unpleasant. |
| Subjective Norm (SN) | Refers to the perceived social pressure to perform or not perform the behaviour ^1^. | People who are important to me think  I should avoid eating high calorie snacks over the next two weeks (disagree strongly/ agree strongly).  People who are important to me think I should eat most of the fish meals served in school (definitely no/ definitely yes). |
| Perceived Behavioural Control (PBC) | A person’s belief as to how easy or difficult performance of the behaviour is likely to be ^1^. | If I want to, I could easily eat high fibre bread for breakfast (definitely yes/ definitely no).  I find limiting my amount of soft drink consumption… very easy/ very difficult. |
| Intention | Represents a person’s plan, decision or self-instruction to perform the behaviour. Intentions are held to indicate how hard people are willing to try, how much of an effort they are planning to exert, in order to perform the behaviour ^2^. | During the next week, I intend to eat 5 servings of fruits and vegetables each day (strongly disagree/ strongly agree).  I intend to have low fat milk for breakfast during the next six months (definitely yes/ definitely no). |
| Food Choice Behaviour | The decisions that individuals take at the point of consumption. Commonly this follows a period of deliberation when deciding between alternative foods. | N/A |
| Health compromising food choice | From the perspective of Temporal Self-Regulation Theory (TST)^3^, health-compromising behaviours, such as the consumption of highly palatable, calorie dense or high-fat foods, are frequently associated with many benefits and few costs at the point of action, whereas the same behaviours are associated with long term costs (e.g. overweight and accompanying illness) and few if any long term benefits. | How many days a week do you drink sugar-containing soft drinks, such as regular cola and sprite (never/seven days per week)?  How many times a week do you consume the following snacks… nuts and potato chips (insert number)? |
| Health promoting food choice | In contrast, health-promoting behaviours, such as the consumption of low calorie, nutrient rich foods, frequently work in the opposite manner, with fewer benefits, and indeed potential costs (e.g. bland or unpleasant taste) at the time of action, but with few costs and many benefits long term (e.g. reduction in risk for contracting chronic conditions such as Coronary Heart Disease). | Over the past 2 weeks, I had (insert number) servings of fruit in a typical day.  Over the past two weeks, I have eaten at least 5 servings of fruit and vegetables (every day/never). |
| Consumption of health promoting food | Dual-process models of behaviour (e.g. ^4,5^) suggest that behaviour is determined by two interacting, parallel systems whereby automatic, impulsive processes, which take input from, amongst other things physiological processes such as hunger are in competition with rational determinants of behaviour such as those described in the TPB.  In this context, the consumption of health promoting foods are held to be governed more by rational processes as they frequently have a lower hedonic value than alternative foodstuffs. For example, simple steamed vegetables are more likely to be chosen for their health-promoting properties (an abstract, rational decision), than for their taste. | I intend to eat an adequate amount of fruit each day (totally agree/ totally disagree).  I intend to have low fat milk for breakfast during the next six months (definitely yes/ definitely no). |
| Avoidance of health compromising food | Dual-process models of behaviour (e.g. ^4,5^) describe how there can be conflict between the two systems, whereby intentions to eat healthily can be overridden by automatic, impulsive responses to consume calorie-dense, palatable food in the immediate environment. In this context, resisting the impulse to consume health compromising foods with high hedonic value may be less likely to be governed by rational determinants of behaviour such as those described in the TPB. | I intend to limit my amount of soft drink consumption (yes definitely/ no definitely not).  I intend to avoid eating high-calorie snacks over the next two weeks (Disagree strongly/ agree strongly). |

**References**

1. Ajzen I, Madden TJ. Prediction of Goal-Directed Behavior - Attitudes, Intentions, and Perceived Behavioral-Control. *J Exp Soc Psychol.* 1986;22(5):453-474.

2. Ajzen I. The Theory of Planned Behavior. *Organ Behav Hum Dec.* 1991;50(2):179-211.

3. Hall PA, Fong GT. Temporal self-regulation theory: A model for individual health behaviour. *Health Psychol Rev.* 2007;1(1):6-52.

4. Hofmann W, Friese M, Wiers RW. Impulsive versus reflective influences on health behavior: a theoretical framework and empirical review. *Health Psychol Rev.* 2008;2(2):111-137.

5. Strack F, Deutsch R. Reflective and impulsive determinants of social behavior. *Pers Soc Psychol Rev.* 2004;8(3):220-247.
