# Supplementary material for: The theory of planned behaviour and discrete food choices: a systematic review and meta-analysis
Source: Int J Behav Nutr Phys Act. 2015 Dec 30;12:162. doi: 10.1186/s12966-015-0324-z (PMC4696173; doi:10.1186/s12966-015-0324-z)
Supplement: Supplementary file 4 [file 12966_2015_324_MOESM4_ESM.docx]

Additional File 4: Forest plots for associations between TRA/TPB variables and behaviour.

Additional Figure 1: Associations between attitude and intention

Additional Figure 2: Associations between subjective norm and intention

Additional Figure 3: Associations between perceived behavioural control and intention

Additional Figure 4: Associations between perceived behavioural control and behaviour

Additional Figure 5: Associations between intention and behaviour
