# Supplementary material for: The theory of planned behaviour and discrete food choices: a systematic review and meta-analysis
Source: Int J Behav Nutr Phys Act. 2015 Dec 30;12:162. doi: 10.1186/s12966-015-0324-z (PMC4696173; doi:10.1186/s12966-015-0324-z)
Supplement: Supplementary file 5 [file 12966_2015_324_MOESM5_ESM.docx]

Additional File 5: Details of method used to measure behaviour in each study.

| Study | Target behaviour | Behaviour measure | No. of items | Sample item |
| --- | --- | --- | --- | --- |
| Aghamolaei et al (2012) | Fish consumption | Self-perception | 1 | How frequently do you eat fish? (Never/Daily) |
| Astrom (2004)/ Astrom & Okullo (2004) | Sugared snack consumption (Time 1) | Self-perception | 2 | During the past three months I have taken sugared snacks and drinks on a daily basis. (Strongly agree/strongly disagree) |
| Sugared snack consumption (Time 2) | Self-perception | 1 | I have taken sugared snacks and drinks on a daily basis during the past weeks. (Strongly agree/strongly disagree) |
| Balian (2008) | Milk consumption | 24-hour dietary recall | 4 | Did you drink milk before school this morning? (Yes/no) |
| Soda consumption | 24-hour dietary recall | 4 | On a usual day during the school week how much soda pop do you drink? (Write the number of cans, cups or 16oz bottles) |
| Berg et al (2000)/ Conner et al (2011) | Milk consumption | 7-day record | N/A | Usual foods for breakfast were precoded and pupils were asked to mark those days on which they had consumed each food. There was also a space for recording other food items. |
| Bread consumption | 7-day record | N/A | Usual foods for breakfast were precoded and pupils were asked to mark those days on which they had consumed each food. There was also a space for recording other food items. |
| Blanchard et al (2009a) | F&V consumption | Self-perception | 6 | During the past week, on average, how many times per day did you eat fruit? |
| Blanchard et al (2009b) | F&V consumption | Self-perception | 2 | Over the past 2 weeks, I had (insert number) ___ servings of fruit in a typical day. |
| Branscum & Sharma (2014) | Snack food consumption | 24-hour dietary recall | N/A | Children were asked to report and record all foods they consumed in the previous 24 hours between breakfast, lunch and dinner. Children were instructed to be as specific as possible for each food and to include important features such as approximate portion sizes and brand names. |
| Brug et al (2006) | Fruit consumption | FFQ | 14 | Respondents were asked about consumption during the previous month. The FFQ included questions about frequency of consumption (from “never/fewer than one time per month” to “7 days a week”) and number of servings (pieces, or small bowls for small fruit such as berries, per day that a certain fruit or juice was consumed). |
| Churchill et al (2008) | High-calorie snack consumption | Self-perception | 8 | How often have you eaten crisps over the past 2 weeks? (Not at all/4 or more times a day) |
| Churchill & Jessop (2011) | High-calorie snack consumption | Self-perception | 8 | How often have you eaten crisps over the past 2 weeks? (Not at all/4 or more times a day) |
| Collins & Mullan (2011) | Snack consumption (Time 1) | Self-perception | 2 | How often do you typically snack in between meals? |
| Snack consumption  (Time 2) | Self-perception | 2 | How often have you snacked over the past week? |
| F&V consumption (Time 1) | FFQ | 7 | How often do you typically eat these foods…fruit/vegetable juice? |
| F&V consumption  (Time 2) | FFQ | 7 | How often do you typically eat these foods…fruit/vegetable juice? |
| Corry (2008) | F&V consumption (Time 1) | Self-perception | 3 | Over the past two weeks, I have eaten at least 5 servings of fruit and vegetables. (Every day/never) |
| F&V consumption (Time 2) | Self-perception | 3 | Over the past two weeks, I have eaten at least 5 servings of fruit and vegetables. (Every day/never) |
| De Bruijn (2010) | Fruit consumption | Self-perception | 10 | How many days per week do you consume the following fruit…apples or pears? |
| De Bruijn et al (2012) | Fruit consumption | Self-perception | 1 | How many days in the past month have you consumed at least two pieces of fruit per day? (None/every day) |
| De Bruijn & Van den Putte (2009) | Soft drink consumption | Self-perception | 2 | How many days per week do you consume sugar-sweetened soft drinks? |
| De Bruijn et al (2009) | Fruit consumption | Self-perception | 8 | How many days per week did you consume the following fruits in the last month… citrus fruits? |
| De Bruijn et al (2007a) | Fruit consumption | Self-perception | 10 | How many days per week do you consume the following fruits… tangerines (Never or less than once per month/seven days per week)? |
| De Bruijn et al (2007b) | Soft drink consumption | Self-perception | 2 | How many days a week do you drink sugar-containing soft drinks, such as regular cola and sprite (never/seven days per week)? |
| De Bruijn et al (2005) | Snack consumption | FFQ | 4 | How many times a week do you consume the following snacks… nuts and potato chips? |
| Karimi-Shahanjarini et al (2012) | Junk food consumption | FFQ | 48 | Snacking was assessed by the consumption of usual snacks consumed by adolescents (26 healthy snacks, 22 unhealthy snacks) during a 1 week period. |
| Kassem (2000)/ Kassem et al (2003) | Milk consumption | FFQ | 10 | How often on average did you drink whole milk during the past year (Never or less than 1 glass per month/more than 3 glasses per day)? |
| Soft drink consumption | FFQ | 12 | How often on average did you drink regular (non-diet) Coke, Pepsi or other cola during the past year (Never or less than 1 glass per month/more than 3 glasses per day)? |
| Kassem & Lee (2004) | Soft drink consumption | FFQ | 12 | How often on average did you drink regular (non-diet) Coke, Pepsi or other cola during the past 12 months (Never or less than 1 glass per month/more than 3 glasses per day)? |
| Kassem & Lee (2005) | Reduced fat milk consumption | FFQ | 10 | How often on average did you drink reduced-fat milk (1% or 2%) during the past 12 months (Never or less than 1 glass per month/more than 3 glasses per day)? |
| Kida & Astrom (1998) | Sugared snack consumption | Self-perception | 4 | During the past six months, how often did you consume chocolate/sweets (Several times a day/seldom or never)? |
| Kim et al (2003) | Dairy product consumption | FFQ | 20 | How often do you usually eat each of the following dairy products…cheese, sliced cheese, cheese spread, including on sandwiches (Never/every day)? |
| Mahon et al (2006) | Ready meals | Self-perception | 1 | How often do you consume ready meals? (Every day or almost every day/never) |
| Masalu & Astrom (2001) | Sugar consumption (Time 1) | Self-perception | 1 | Think about the three last months – how often have you tried to avoid between-meal intake of sugared snacks and drinks? (Very often/never) |
| Sugar consumption (Time 2) | Self-perception | 1 | During the past few weeks I have tried to avoid between-meal intake of sugared snacks and drinks. (Strongly agree/strongly disagree) |
| Mitterer-Daltoe et al (2013) | Fish consumption | Self-perception | 1 | How frequently do I eat fish? (Daily/never) |
| Murnaghan et al (2010) | F&V consumption | Self-perception | 1 | During the last 30 days, I ate at least five servings of fruits and vegetables on (insert a number between 0 and 30) days. |
| O’Neal et al (2014) | F&V consumption | Self-perception | 2 | How many servings of fruits and vegetables do you usually eat each day (zero or one servings per day on average/five or more servings per day on average). |
| Onwezen et al (2014) | Fruit consumption | FFQ | 8 | How often did you consume tangerines in the past week? (Not at all/every day) |
| Povey et al (2000) | F&V consumption | FFQ | 63 | How often do you eat each of the following foods… bananas? (2 or more times a day/rarely-never) |
| Prell et al (2002) | Fish consumption | Observation of behaviour | N/A | Consumption of fish was measured by structured observations in the school canteen setting. One person observed the taken fish component on each pupil’s plate and another person the leftovers. The observations aimed to categorize the pupils into non-eaters, tasters and eaters of fish. Participants who had eaten more than half a portion on at least two of the four measurement occasions were categorised as eaters. All those who has taken fish at least once at the four lunches were categorised as tasters. |
| Richetin et al (2008) | Soft drink consumption | Self-perception | 1 | Participants were asked about their last week consumption of a series of fizzy soft drinks (e.g. coke) expressed in units (e.g. a can of 330ml equals one unit). Participants were asked to report how many in each category they drank during the last week. |
|  | Observation of behaviour | N/A | Soft drink consumption was measured by weighing the amount of the drink each participant drunk (i.e. the difference between the weight at the beginning and the weight after) during a taste test). |
| Sharifirad et al (2013) | Fast food consumption (Time 1) | Self-perception | 2 | On average, how often do you eat fast food? (never/more than once per day) |
| Fast food consumption (Time 2) | Self-perception | 8 | In the last week, how many times have you eaten pizza? |
| Sjoberg et al (2012) | Wholegrain bread consumption | FFQ | 8 | Each time you eat wholegrain sandwich bread how much do you usually eat? (1 slice/more than 2 slices) |
| Tak et al (2011) | Sugar-sweetened beverage consumption | Self-perception | 2 | How many days a week do you usually drink sugar-sweetened (not “light” or “diet”) beverages? |
| Tak et al (2013) | Fruit consumption | Self-perception | 2 | On how many days per week did you consume fruit in the last month? |
| Towler & Shepherd (1991) | Chip consumption | Self-perception | 1 | Behaviour was assessed with a single question. Subjects responded using a seven point scale which ranged from never consuming chips through to consuming chips once a day. |
| Tuu et al (2008) | Fish consumption | Self-perception | 1 | How many times on average during the last year have you eaten fish as a meal? (9-12 times or more a week/never) |
| Verbeke & Vackier (2005) | Fish consumption | Self-perception | 1 | How frequently do you eat fish? (Daily/never) |
| Verplanken (2006) | Snack consumption | Self-perception | 1 | How many times have you eaten snack items in the past 7 days? (Not at all/more than 14 times) |
|  | 7-day record | N/A | Participants were instructed to write down all food items they consumed outside regular meals for the next 7 days. |
| Zoellner et al (2012) | Sugar-sweetened beverage consumption | FFQ | 19 | A 19 item beverage intake questionnaire was used to assess beverage intake over the past month. The instrument queries 7 frequencies, including: never or less than 1 time/wk, 1 time/wk, 2 or 3 times/wk, 4-6 times/wk, 1 time/d, 2 times/d, or 3 times or more/d. Portion sizes are also reported in fluid ounces and incluse less than 6,8,12,16, or more than 20. Sugar-sweetened beverage intake is quantified by summing kilocalories from 7 items including regular soft drinks, sweetened juice beverage/drink, sweetened tea, coffee with sugar, mixed alcoholic drinks, meal replacement shakes/protein drinks, and energy drinks. |
